# A Survey Using High-Throughput Sequencing Suggests That the Diversity of Cereal and Barley Yellow Dwarf Viruses Is Underestimated

**DOI:** 10.3389/fmicb.2021.673218

**Published:** 2021-05-11

**Authors:** Merike Sõmera, Sébastien Massart, Lucie Tamisier, Pille Sooväli, Kanitha Sathees, Anders Kvarnheden

**Affiliations:** ^1^Department of Chemistry and Biotechnology, Tallinn University of Technology, Tallinn, Estonia; ^2^Laboratory of Integrated and Urban Phytopathology, Gembloux Agro-Bio Tech – University of Liège, Gembloux, Belgium; ^3^Department of Plant Protection, Estonian Crop Research Institute, Jõgeva, Estonia; ^4^Department of Plant Biology, Swedish University of Agricultural Sciences, Uppsala, Sweden; ^5^Institute of Agricultural and Environmental Sciences, Estonian University of Life Sciences, Tartu, Estonia

**Keywords:** HTS, *Luteovirus*, BYDV, CYDV, diagnostics, wheat, epidemiology, OYV

## Abstract

Worldwide, barley/cereal yellow dwarf viruses (YDVs) are the most widespread and damaging group of cereal viruses. In this study, we applied high-throughput sequencing technologies (HTS) to perform a virus survey on symptomatic plants from 47 cereal fields in Estonia. HTS allowed the assembly of complete genome sequences for 22 isolates of cereal yellow dwarf virus RPS, barley yellow dwarf virus GAV, barley yellow dwarf virus PAS (BYDV-PAS), barley yellow dwarf virus PAV (BYDV-PAV), and barley yellow dwarf virus OYV (BYDV-OYV). We also assembled a near-complete genome of the putative novel species BYDV-OYV from Swedish samples of meadow fescue. Previously, partial sequencing of the central part of the coat protein gene indicated that BYDV-OYV represented a putative new species closely related to BYDV-PAV-CN, which currently is recognized as a subtype of BYDV-PAV. The present study found that whereas the 3′gene block of BYDV-OYV shares the closest relationship with BYDV-PAV-CN, the 5′gene block of BYDV-OYV shows the closest relationships to that of BYDV-PAS. Recombination detection analysis revealed that BYDV-OYV is a parental virus for both. Analysis of complete genome sequence data indicates that both BYDV-OYV and BYDV-PAV-CN meet the species criteria of genus *Luteovirus*. The study discusses BYDV phylogeny, and through a systematic *in silico* analysis of published primers for YDV detection, the existing gaps in current diagnostic practices for detection of YDVs, proposing primer pairs based on the most recent genomic information for the detection of different BYDV species. Thanks to the rising number of sequences available in databases, continuous updating of diagnostic primers can improve test specificity, e.g., inclusivity and exclusivity at species levels. This is needed to properly survey the geographical and host distribution of the different species of the YDV complex and their prevalence in cereal/barley yellow dwarf disease epidemics.

## Introduction

Yellow dwarf viruses (YDVs) of the family *Luteoviridae* constitute a complex of ssRNA viruses that are the most widespread group of cereal viruses worldwide (a list of the countries with recorded entries can be found at https://www.cabi.org/isc/datasheet/10539). YDVs are transmitted by more than 25 aphid species in a persistent non-propagative manner (Halbert and Voegtlin, 1995). Perennial grasses play an important role in the epidemiology of YDVs as more than 150 potential reservoir host species have been recorded in the family Poaceae (Gramineae; D’Arcy, 1995). Yield losses of 13–45 kg ha^–1^ for each 1% increase in YDV incidence are reported, ranging up to 80% of a total yield. The actual losses are dependent on symptom severity, which depends on the particular YDV species infecting the crop plants and on the varying transmission efficiency of YDVs by different vector species (Van den Eynde et al., 2020).

The aphid transmission properties of polero- and luteoviruses are determined by their capsids, which share a common evolutionary origin (Martin et al., 1990). Currently, in the genus *Polerovirus*, viruses of five recognized species have been found infecting cereals or grasses: *Cereal yellow dwarf virus RPV*, *Cereal yellow dwarf virus RPS* (CYDV-RPS), *Maize yellow dwarf virus RMV* (MYDV-RMV), *Maize yellow mosaic virus* (MaYMV), and *Sugarcane yellow leaf virus*. In addition, recent reports have indicated two novel tentative members of the genus *Polerovirus*: Barley virus G (BVG; Zhao et al., 2016) and Wheat leaf yellowing-associated virus (Zhang et al., 2017). In the genus *Luteovirus*, there are five species with members infecting cereals or grasses: *Barley yellow dwarf virus PAV* (BYDV-PAV), *Barley yellow dwarf virus PAS* (BYDV-PAS), *Barley yellow dwarf virus MAV*, *Barley yellow dwarf virus kerII*, and *Barley yellow dwarf virus kerIII*. Currently, Barley yellow dwarf virus GAV (BYDV-GAV) is considered as a subspecies of *Barley yellow dwarf virus MAV*, and Barley yellow dwarf virus PAV-CN is considered as a subspecies of *Barley yellow dwarf virus PAV*. Partial sequencing of the coat protein (CP) gene suggested a putative novel cereal-infecting luteovirus Barley yellow dwarf virus OYV (BYDV-OYV), whose taxonomic status remained unclear because of the lack of a complete genome sequence (Bisnieks et al., 2004). Two established species, Barley yellow dwarf virus GPV (BYDV-GPV) and Barley yellow dwarf virus SGV (BYDV-SGV), have not been assigned to either genus yet.

In the current study, we identified and sequenced the genome of BYDV-OYV for isolates from spring wheat and oat samples collected in Estonia and from meadow fescue samples collected in Sweden. In addition to BYDV-OYV, we also detected BYDV-PAS, BYDV-PAV, BYDV-GAV, and CYDV-RPS in a field survey in Estonia using high-throughput sequencing (HTS). Interestingly, half of these species have been reported seldomly. Combining the genome sequences generated during this study and the available sequences in the database, we propose also new diagnostic primers for specific detection of the BYDV different species.

## Materials and Methods

### Plant Material

In 2012–2015, the leaf samples of cereal plants (wheat, barley, oat, rye, and triticale) showing chlorotic mottle or stripes, yellowing, reddening, stunting, or other symptoms characteristic of possible viral infection were collected from 47 fields, mainly located in south-eastern Estonia (Sõmera et al., 2020). Two samples of meadow fescue (*Festuca pratensis*) that were analyzed in this study originated from Sweden (Lit, County of Jämtland) and were collected in 2010.

### Characterization of Swedish BYDV-OYV Isolate Using IC-RT-PCR and Sanger Sequencing

Extracts of two meadow fescue (*F. pratensis*) plants were used as the source of viral RNA in immunocapture (IC) RT-PCR (Bisnieks et al., 2004) with polyclonal antibodies for BYDV-PAV (Loewe Biochemica). Initially, RT-PCR was carried out using the luteovirus universal primer pair Shu-F and Yan-R (Malmstrom and Shu, 2004) to amplify the region corresponding to ORF4. The other genomic regions from ORF1 to 3′untranslated region (UTR) were amplified using a combination of published BYDV-PAV-CN (Liu et al., 2007) and newly designed BYDV-OYV primers: P326(+)/P12(–), P115(+)/OYV5(−), OYV4(+)/OYV1(−), OYV2(+)/OYV3(−), and P47(+)/P55(−; Table 1). cDNA was synthesized using the corresponding reverse primer and Superscript III reverse transcriptase (Invitrogen) according to the manufacturer’s instructions, while DreamTaq DNA polymerase was used for PCR. The amplification conditions were the following: 95°C for 2 min, followed by 35 cycles of 95°C for 30 s, 48–63°C for 2 min, 72°C for 1–2 min, with a final extension at 72°C for 10 min. As a negative control, PCR was run without cDNA template. Purified amplification products were ligated into pJET1.2 vector (Invitrogen) and transformed into *Escherichia coli* DH5α competent cells. Two clones of each amplification product were sequenced at Macrogen Inc., Amsterdam. A genomic sequence was assembled from the overlapping PCR fragments using MegaAlign.

**TABLE 1:** Primers used in the current study.

Primers	Nucleotide sequence 5′ to 3′	Expected position	References
P326(+)	GACTTCGAGGCNGANCTCGCT	330–350	Liu et al., 2007
P12(–)	GCTCCGTCTGTGACCGCAAT	1226–1245	Liu et al., 2007
P115(+)	GGGTTTTTAGAGGGGCTCTGT	1157–1177	Liu et al., 2007
OYV5(–)	TCACCATGTTGAAGCCGTATT	2199–2219	This study
OYV4(+)	ATGTTCGTTGAGGATAAGATGC	1973–1994	This study
OYV1(–)	AGTACGTGAGAGCTAATGTAC	2800–2820	This study
Shu-F	TACGGTAAGTGCCCAACTCC	2650–2669	Malmstrom and Shu, 2004
Yan-R	TGTTGAGGAGTCTACCTATTTG	3459–3480	Malmstrom and Shu, 2004
OYV2(+)	TGAACTCGACACTGCGTGCA	3237–3256	This study
OYV3(–)	CTACCCGAGCTTATGAACCT	4857–4876	This study
P47(+)	GCAAAGGAGTACAAGGCACAAT	4777–4798	Liu et al., 2007
P55(–)	GGATTGCTATGGTTTATGTCC	5491–5511	Liu et al., 2007
OYV5pR1	AAGTCCGTCCAAGCCTCGG	533–541	This study
OYV5pR2	CTTTGACGCTGGCTCCAATGAGC	161–183	This study
PasF	GAAGAGGGCCAAATTCTATACC	3003–3024*	This study
OyvF	CCAATTCCTCAGGGATCC	3080–3097	This study
GavF	GTTACAAGATCACAAACGTCAAG	3156–3178**	This study
PavF	CTTCACAATCAGCAGGAC	3261–3278***	This study

Primer binding positions are shown respective to full length sequence of BYDV-OYV Avinurme2 isolate (MK012645; unless mentioned otherwise). *BYDV-PAS (MK012660), **BYDV-GAV (MK012663), and ***BYDV-PAV (MK012661).

### 5′ RACE Reaction for BYDV-OYV Genome

Total RNA was extracted from frozen plant material using Trizol reagent (Invitrogen) according to the manufacturer’s protocol. The 5′ RACE reactions were performed using the Roche 5′/3′ RACE kit (ver. 13) for Avinurme2 and Ulvi samples from Estonia. The OYV5pR1 primer (Table 1) directed toward the 5′end of the BYDV-OYV genome was used for primer extension in RT-reaction and later on after polyA-tailing reaction in PCR together with the oligoT-anchoring primer. Next, the OYV5pR2 primer (Table 1) was used for nested PCR together with the oligoT-anchoring primer. RT-PCR products were separated by gel electrophoresis. The RT-PCR product of 183 bp was purified from the gel using GeneJET Gel Extraction and DNA Cleanup kit, ligated into the pJET1.2 blunt vector (all Thermo Scientific), and transformed into *E. coli* DH5α competent cells. Plasmid DNA extracted from three individual colonies was sequenced using a vector-specific primer.

### Sequencing of Small RNA Libraries and Virus Identification

The total RNA of 1–7 individual plant samples collected from the same field were pooled in equal concentrations to synthesize the indexed HTS libraries using the TruSeq siRNA kit (Illumina) according to the Sample Preparation Guide 02/2013. Depending on the number of collected plant samples, 1–3 HTS libraries were prepared for each field. The libraries were sequenced on HiSeq2500 as described in Sõmera et al. (2020). The number of raw reads varied between the library samples from 4.5 to 17.2 million. *De novo* contig assembly was done using the Oases 0.2.08 software (Schulz et al., 2012) varying the k-mer value from 15 to 31. The assembled contigs were analyzed using the BLAST + version 2.2.28 against the GenBank non-redundant databases of nucleotide collection and protein sequences, respectively, by using BlastN and BlastX search with standard parameters. All HTS data libraries were mapped to reference genomes using Geneious Prime software (2019.0.4). For that, 90 luteovirus, 10 polerovirus, BYDV-SGV, and MYDV-RMV complete genome sequences publicly available in March 2018 were retrieved from NCBI GenBank (see column 1 in Supplementary Table 1). *De novo* assembled complete genome consensus sequences of BYDV-OYV Avinurme2 and Ulvi isolates were used as the reference sequences for other BYDV-OYV sequences.

Mapping against the closest reference generated a consensus sequence. If any gaps existed in the sequence that was not filled during re-mapping, the mapping against other isolates was checked and in case the gaps were covered with mapped reads, the gaps were filled accordingly. This combined consensus was used as a draft reference sequence to repeat the mapping to obtain the final consensus sequence. The assembled complete consensus genome sequences were annotated and deposited in GenBank. Incomplete consensus sequences were used only for the identification of the virus species. After assembly of the complete genome consensus sequences, open reading frames (ORFs) were identified using the “Annotate and Predict” function in the Geneious program. The parameters were set for the standard genetic code using a minimum of 100 codons, starting with the AUG initiation codon. For detection of the non-AUG start codon of ORF3a, the near-cognate codons AUU, ACG, AUA, or CUG were considered. The site for -1 programmed ribosomal frameshift was identified according to alignment with other luteoviruses.

### Pairwise Identity Calculations and Phylogenetic Analysis

The identity calculations of nucleotide sequences of the complete genome sequences and individual protein sequences were performed using the MUSCLE multiple sequence alignment tool implemented in the Geneious Prime program. The maximum-likelihood tree of nucleotide sequences for luteoviruses was constructed using PhyML ver. 3.1, implemented in the Seaview 4.6.1 program, with a GTR nucleotide substitution mode and 1000 bootstrap replications (Gouy et al., 2010).

### Recombination Detection Point Analysis of BYDV-OYV

Recombination analysis of Estonian YDV isolates was carried out using the Recombination Detection Program (RDP4; Martin et al., 2015) with a selection of reference luteovirus sequences aligned by ClustalW in MegaAlign (DNASTAR Lasergene 11). The recombination analyses were done as described in Kamali et al. (2016).

### Design of Species-Specific Primers Able to Discriminate Between BYDV-PAV, -GAV, -PAS, and -OYV

MUSCLE multiple sequence alignment of luteovirus full-length genomes retrieved from GenBank was used for the selection of unique primer binding sites for BYDV-PAV, -GAV, -PAS, and -OYV in the region of ORF3/ORF4. A mix of new species-specific forward primers PasF, OyvF, GavF, and PavF (Table 1) together with the universal primer Yan-R (Malmstrom and Shu, 2004) was tested for the detection of single, double, triple, or quadruple infection of BYDV-PAV, -GAV, -PAS, and -OYV. The field-collected high-throughput sequenced BYDV samples were used as a reaction template for each species. Non-infected plant material was used as a negative control. Total RNA (1 μg) was used as a template in single-template RT-PCR. Per 20 μl reaction volume, 1 μl of dNTP mix (10 mM), 1 μl of Yan-R primer (200 μM), 200 U of Maxima reverse transcriptase, and 40 U of Ribolock RNase inhibitor (both Thermo Scientific) were used. Reverse transcription was carried out at 50°C for 1 h. In PCR, 0.5 μl of synthesis product was used as a template. The detection of multiple templates (BYDV-PAV, -GAV, -PAS, and -OYV) was tested using artificial mixes of their first-strand synthesis products (0.5 μl of each). The reaction was carried out using DreamTaq DNA polymerase green mix together with the primer mix of Yan-R (1 μl of 200 μM stock), PavF, GavF, PasF, and OyvF primers (0.5 μl each of 200 μM stocks). Yan-R primer was added after 5 cycles to favor the initial amplification of different BYDV templates. The amplification conditions were the following: 95°C for 3 min, followed by 35 cycles of 95°C for 1 min, 52°C for 1 min, 72°C for 1 min, with a final extension at 72°C for 5 min. Reaction products were separated electrophoretically, purified from the gel, and sequenced to verify the viral origin.

### *In silico* Primer Binding Tests

*In silico* binding tests were performed to evaluate the specificity and sensitivity of 32 primer pairs (Robertson et al., 1991; Balaji et al., 2003; Malmstrom and Shu, 2004; Nagy et al., 2006; Deb and Anderson, 2008; Zhao et al., 2010; Tao et al., 2012; Svanella-Dumas et al., 2013) commonly used to detect YDVs species, including four newly designed pairs from this study (Supplementary Table 2). All primers were tested against 112 BYDV genomes (see column 1 in Supplementary Table 1) using the “Test with Saved Primers” option in Geneious. A maximum number of 0 to 3 mismatches were allowed between the primers and the viral sequences. Two measures were performed to assess the binding efficiency of each primer pair. First, the sensitivity was calculated as a ratio between the number of sequences of one BYDV species correctly identified and the total number of BYDV sequences belonging to this species. In this analysis, BYDV-PAS isolates 064, 0109, and KS-SHKR, which yet are identified as BYDV-PAV in GenBank, were treated as BYDV-PAV. Second, the specificity was calculated as a ratio between the number of sequences correctly not identified and the total number of BYDV sequences that should not have been identified. To be close to real PCR conditions, a mismatch on the last base of the 3′ end primers is considered to be an absence of binding.

## Results

### Virus Identification by the Analysis of HTS Data

The analysis of HTS results from the virus survey in Estonia revealed the presence of one polerovirus, CYDV-RPS, and four luteoviruses, BYDV-GAV, BYDV-PAV, BYDV-PAS, and the tentative new species BYDV-OYV, in 24 of the 47 cereal fields included in the survey. A summary of the mapping results of specific HTS libraries is shown in Table 2. A near-complete consensus sequence of CYDV-RPS (MK012664) with a few small gaps was obtained from one HTS library. A consensus sequence of BYDV-PAV (MK012661) was also obtained from one HTS library. BYDV-GAV was detected from three HTS libraries for samples collected in different locations and during several years. Consensus sequences covering the complete genome were obtained for the Jõgeva3 and Kumna isolates (MK012662 and MK012663, respectively), but not for the Abja isolate (57.4% genome coverage). BYDV-PAS was detected in 12 HTS libraries. The full genome was assembled for 11 samples (MK012650-MK012660), while the genome sequence of the isolate BYDV-PAS Listaku was recovered only partially (19.6% of the full genome sequence). Detection of BYDV-OYV was confirmed in seven samples collected during three different years and in several locations. Complete consensus genome sequences of BYDV-OYV were obtained for seven isolates (MK012643-MK012649). The genomes of the isolates Avinurme2 and Ulvi were assembled *de novo*. The five other consensus genomes of BYDV-OYV were obtained by mapping these two references.

**TABLE 2:** Identification of YDVs collected in Estonia during the current study.

Field no	YDV species identified	Isolate name	GenBank Acc. No.	Genome coverage depth, average	Virus specific read/total reads in the library
1	BYDV-PAS	Imavere	MK012660	822.8	193,297/3,054,788
2	BYDV-PAS	Listaku	–		
3	BYDV-PAS	Jõgeva1	MK012659	156.6	37,484/4,298,886
4	BYDV-PAS	Jõgeva2	MK012658	197.3	49,267/3,362,095
5	BYDV-PAS	Matapera	MK012657	535.0	132,644/6,000,966
8	BYDV-PAS	Puide	MK012656	158.9	35,129/3,090,120
9	BYDV-GAV	Kumna	MK012663	434.4	111,635/2,304,733
10	BYDV-GAV	Jõgeva3	MK012662	315.5	79,589/10,679,704
11	BYDV-PAS	Jõgeva4	MK012654	359.2	91,136/1,220,020
14	BYDV-PAS	Väimela	MK012655	103.4	24,760/9,172,512
17	CYDV-RPS	Olustvere1-O	MK012664	24.2	6,129/4,998,325
18	BYDV-PAV	Olustvere1-B	MK012661	225.4	53,606/6,340,305
19	BYDV-PAS	Olustvere2-B	MK012653	383.3	86,751/5,531,072
20	BYDV-PAS	Olustvere2-W	MK012652	481.4	121,789/5,339,363
23	BYDV-PAS	Rannu1	MK012651	494.0	118,588/7,259,253
26	BYDV-OYV	Rannu2	MK012649	260.0	63,991/6,056,844
30	BYDV-OYV	Õru	MK012648	81.5	18,530/8,529,024
32	BYDV-PAS	Põlva	MK012650	61.8	15,999/2,863,991
35	BYDV-GAV	Abja	–		
39	BYDV-OYV	Ulvi	MK012647	286.9	72,813/7,931,998
40	BYDV-OYV	Avinurme1	MK012646	244.7	63,453/3,070,516
41	BYDV-OYV	Avinurme2	MK012645	371.8	86,326/8,693,892
43	BYDV-OYV	Avinurme3	MK012644	237.7	57,958/6,163,353
47	BYDV-OYV	Saunja	MK012643	465.7	120,362/6,946,669

### Characterization of BYDV-OYV Genome

**FIGURE 1:**
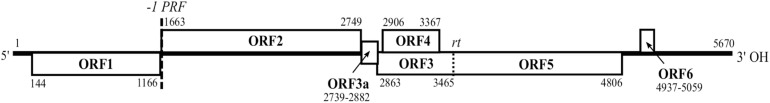
Genome organization of BYDV-OYV. The nucleotide positions of open reading frames (ORFs) are exemplified using Avinurme2 isolate (MK012645). The functions of ORFs are assumed to be analogous to those assigned in other luteoviruses: P1 protein is encoded by ORF1, RdRp is translated via -1 programmed ribosomal frameshift (-1 PRF) and encoded by ORF2, P3a protein is encoded by ORF3a, viral coat protein (CP) is encoded by ORF3, viral cell-to-cell movement protein (MP) is encoded by ORF4, read-through domain (RTD) is encoded by ORF5 and translated via ORF3 stop codon read-through (rt), P6 is encoded by ORF6. Subgenomic RNAs (sgRNA) are needed for translation of P3a, MP, CP, or CP-RTD and P6.

Using 5′RACE sequencing, the 5′ ends of the complete genomes assembled by HTS data analysis were confirmed (100% identity) for two Estonian isolates (Avinurme2 and Ulvi) of the new candidate species. In parallel, BYDV-OYV was identified by IC-RT-PCR using BYDV-PAV antibodies in two meadow fescue samples collected in Sweden. Using the strategy of primer walking, 5,133 nt of the Swedish isolate (MK012642) was sequenced. The obtained sequence of the Swedish isolate covered 90% of the genome length, lacking the 5′-UTR, the beginning of ORF1, and a part of 3′UTR. The complete genome sizes for the seven Estonian BYDV-OYV isolates ranged between 5,664 and 5,681 nt (MK012643- MK012649). The biggest difference for the variation in genome length between the Estonian isolates lies at the beginning of ORF5 [encoding the read-through domain; read-through domain protein (RTD)] where the isolates of Saunja and Rannu have an insertion of 18 nucleotides and the isolates Ulvi, Avinurme1, Avinurme2, and Avinurme3 have an insertion of 6 nucleotides compared to the isolates from Sweden and Õru. The Õru isolate has an additional insertion of a single nucleotide within the 3′-UTR. The Estonian BYDV-OYV isolates sampled from cereal plants shared a nucleotide identity of 93.9–98.6% with each other and showed an identity of 92.7–93.4% with the Swedish BYV-OYV isolate. The identity of the new sequences with the previously sequenced partial 502-bp CP gene sequence fragment of BYDV-OYV (Latvian isolate; AJ563410) was 95.8–97.0%. Assembly of the complete genome sequences for BYDV-OYV isolates revealed a genome organization and size characteristic of luteoviruses (Figure 1).

The sequence of BYDV-OYV Avinurme2 (MK012645) was further used for detailed genome annotation. The length of the BYDV-OYV Avinurme2 genome is 5,670 nt, with 5′- and 3′-UTRs of 143 and 611 nt, respectively. The beginning of the 5′-UTR is conserved between isolates of BYDV-OYV, but slightly different from other BYDVs, being most close to BYDV-PAS isolates. The 3′-UTR ends with the conserved motif CGGCAUCCC, which is also characteristic of BYDV-PAS and BYDV-PAV-CN. The coding region between the terminal UTRs is polycistronic and consists of seven predicted ORFs.

The ORF1 of BYDV-OYV (nt position 144–1166) encodes a protein P1 with a calculated molecular weight of 38.8 kDa. In case of the event of -1 programmed ribosomal frameshifting at the GGGUUUU just before the ORF1 stop codon, translation can continue from nt position 1163 in the frame of ORF2 until a stop codon at nt position 2747–2749, leading to the synthesis of a 98.8 kDa polyprotein P1-P2 constituting the viral RNA-dependent RNA polymerase (RdRp). Translation of ORF3a is predicted to depend on non-AUG initiation (Smirnova et al., 2015). Here, we identified the near-cognate codon ACG at nt position 2739–2741 and a stop codon at nt position 2880–2882. The calculated molecular weight for BYDV-OYV P3a is 5.3 kDa. The ORFs 3 and 4 overlap in different reading frames. BYDV-OYV ORF3 encodes a viral CP of 22 kDa; it starts at nt position 2863 and ends with an amber stop codon at nt position 3463–3465. The viral aphid transmission factor which is also called the RTD is translated from ORF5 as a polyprotein CP-RTD with a calculated molecular weight of 71.2 kDa via a read-through of the ORF3 stop codon. The sequence context (AAAUAGGUAGAC) around the ORF3 amber stop codon is conserved. BYDV-OYV ORF4 (nt pos. 2906–3367) encodes the cell-to-cell movement protein P4 with a calculated molecular mass of 17 kDa. Before the beginning of ORF6, there is an internal UTR of 131 nt. ORF6 of BYDV-OYV lies in nt position 4937–5059. The calculated molecular weight of the putative P6 protein is 4 kDa.

### Phylogenetic Grouping of BYDV Species

**FIGURE 2:**
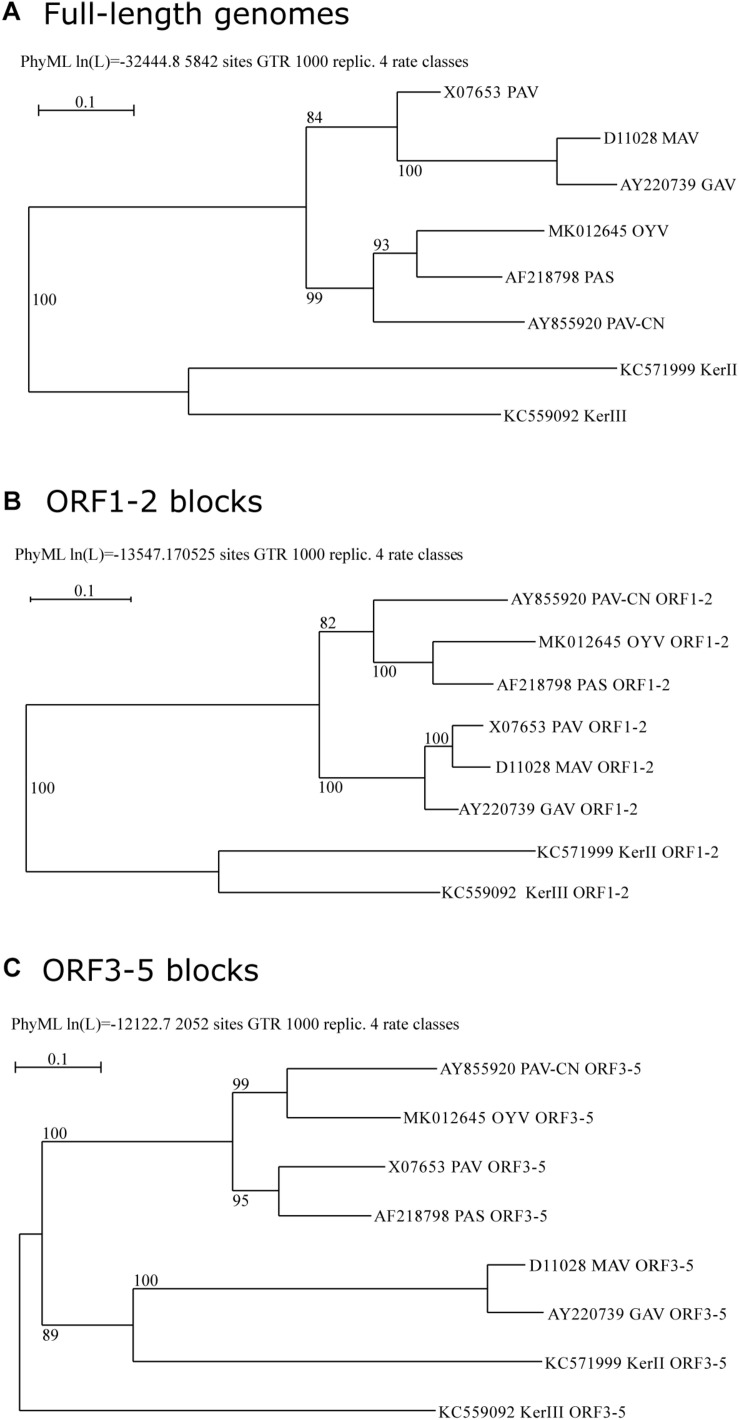
Maximum-likelihood phylogenetic trees of BYDVs belonging to the genus *Luteovirus* based on a MUSCLE multiple sequence alignment of the complete genome **(A)**, ORF1-2 **(B)**, or ORF3-5 **(C)** nucleotide sequences, respectively. The trees were constructed using PhyML ver. 3.1, implemented in the Seaview 4.6.1 program, with a GTR nucleotide substitution model. Percentage values on the tree indicate the frequency with which the grouping occurred after bootstrapping with 1000 replicates and is shown only when >70. Scale bars indicate the distance in nucleotide substitution per site.

Phylogenetic analyses of BYDV genomes supported clustering of cereal-infecting BYDVs (Figure 2A). Within this group, BYDV-PAV and BYDV-MAV (and its subspecies BYDV-GAV) grouped with 84% of bootstrap support, and BYDV-PAV-CN, BYDV-PAS, and BYDV-OYV grouped with 99% support whereas BYDV-OYV was closer to BYDV-PAS than to BYDV-PAV-CN. Phylogenetic analyses of ORF1-2 and ORF3-5 gene blocks (encoding P1-RdRp and CP-RTD, respectively; Figures 2B,C) showed conflicting results with each other and with the genome tree, a characteristic of recombination-driven species evolution. BYDV-OYV ORF1-2 clustered with that of BYDV-PAS and BYDV-PAV-CN, being closer to BYDV-PAS ORF1-2 (100% of support). BYDV-OYV ORF3-5 clustered with BYDV-PAV-CN ORF3-5 (99% of support) whereas BYDV-PAS ORF3-5 clustered with 95% of bootstrap support to that of BYDV-PAV. ORF3-5 of BYDV-MAV (and its subspecies BYDV-GAV) was distant to that of other cereal-infecting BYDVs, clustering with barley yellow dwarf kerII (BYDV-KerII) ORF3-5 (89% of support).

### Pairwise Identity Between the Genomes and Respective Gene Products

Overall genome identity is higher for BYDV-OYV and BYDV-PAS (82.8%) than for BYDV-OYV and BYDV-PAV-CN (79.4%). Between BYDV-PAV-CN and BYDV-PAS, the overall genome identity is 79.7% (Table 3). Phylogenetic analyses of the ORF1-2 and ORF3-5 gene blocks are in concordance with their gene product identity percentages. Comparison of BYDV-OYV and BYDV-PAV-CN indicates that whereas proteins encoded by the 3′ half of their genomes (MP, CP, and RTD) share the highest identity, the proteins (P1, RdRp) encoded by the 5′ part of the BYDV-OYV genome are most similar to those of BYDV-PAS (Table 3). RdRp, encoded in the 5′ gene block, showed an amino acid identity of 90.5% between BYDV-OYV and BYDV-PAV-CN, but 94.9% between BYDV-OYV and BYDV-PAS, and 91.5% between BYDV-PAV-CN and BYDV-PAS. CP, encoded in the 3′ gene block, showed an identity of 88.0% between BYDV-OYV and BYDV-PAV-CN, but 71.5% between BYDV-OYV and BYDV-PAS, and 72.5% between BYDV-PAV-CN and BYDV-PAS. The short and highly conserved P3a protein (encoded from ORF3a located between the ORF1-2 and ORF3-5 gene blocks) showed the overall highest identities: 93.6% between BYDV-OYV and BYDV-PAV-CN, but 95.7% between BYDV-OYV and BYDV-PAS, and 97.9% between BYDV-PAV-CN and BYDV-PAS.

**TABLE 3:** Nucleotide and amino acid percentage identity calculations based on Muscle multiple sequence alignments of the genome sequences and gene products encoded by BYDV-OYV (MK012645) or BYDV-PAV-CN (AY855920) and the respective sequences of BYDV-KerII (KC571999), BYDV-KerIII (KC571992), BYDV-MAV (D11028), BYDV-GAV (AY220739), BYDV-PAS (AF218798), and BYDV-PAV (X07653).

BYDV-OYV	BYDV-KerII	BYDV-KerIII	BYDV-MAV	BYDV-GAV	BYDV-PAS	BYDV-PAV	BYDV-PAV-CN
Genome	60.9	64.1	69.1	70.5	82.8	76.1	79.4
P1	52.1	51.7	77.6	77.3	89.7	77.6	77.3
RdRp	75.8	77.0	87.9	87.1	94.9	88.1	90.5
P1-RdRp	66.5	67.6	83.9	82.9	92.9	84.0	85.4
P3a	78.7	76.6	95.7	91.5	95.7	91.5	93.6
MP	69.3	66.2	74.0	73.4	79.1	77.8	85.6
CP	63.0	58.5	66.0	66.0	71.5	75.5	88.0
CP-RTD	56.7	57.4	60.2	60.1	81.7	80.8	83.5
RTD	54.3	57.5	57.4	57.6	86.7	83.8	81.9
P6	37.8	ND	30.5	30.5	38.5	27.0	55.0

**BYDV-PAV-CN**	**BYDV-KerII**	**BYDV-KerIII**	**BYDV-MAV**	**BYDV-GAV**	**BYDV-PAS**	**BYDV-PAV**	**BYDV-OYV**

Genome	60.9	63.8	69.8	72.0	79.7	76.1	79.4
P1	53.7	52.3	79.1	79.9	80.5	79.4	77.3
RdRp	75.0	77.3	86.7	86.4	91.5	86.7	90.5
P1-RdRp	66.7	67.8	83.7	83.5	87.2	83.9	85.4
P3a	78.7	76.6	93.6	93.6	97.9	93.6	93.6
MP	69.3	66.2	72.1	71.4	79.1	77.8	85.6
CP	64.5	57.5	71.0	70	72.5	74.5	88.0
CP-RTD	56.2	57.6	59.7	59.9	77.6	77.3	83.5
RTD	54.9	59.3	57.6	57.4	80.5	79.2	81.9
P6	32.4	ND	45.0	42.5	38.5	28.6	55.0

### Recombination Detection in BYDV-OYV Genome

**FIGURE 3:**
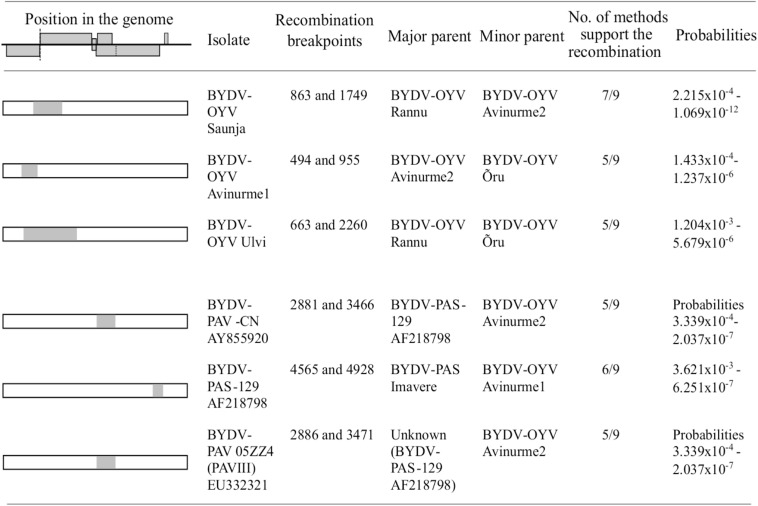
Determination of putative recombination events between the isolates of BYDV-OYV; and BYDV-OYV, BYDV-PAS, and BYDV-PAV-CN using RDP4 software. Only events supported by minimum of five methods are reported. Nucleotide numbering corresponds to the aligned sequences.

Recombination detection tests (Figure 3) for putative recombination events in the BYDV-OYV genome detected intra-specific recombination in the genome of isolates Saunja, Avinurme 1, and Ulvi. When compared with representative genomes of other BYDV species, BYDV-OYV was also identified as a putative minor parent for recombinant regions in the genomes of BYDV-PAV-CN (AY855920 and EU332321) and BYDV-PAS-129 (AF218798) with two different BYDV-PAS genotypes as a putative major parent, respectively. In all these cases, detection of recombination was supported by at least 5 out of 9 methods used.

### New Multiplex-RT-PCR for Discrimination of BYDV-OYV, -PAS, -PAV, and -GAV

**FIGURE 4:**
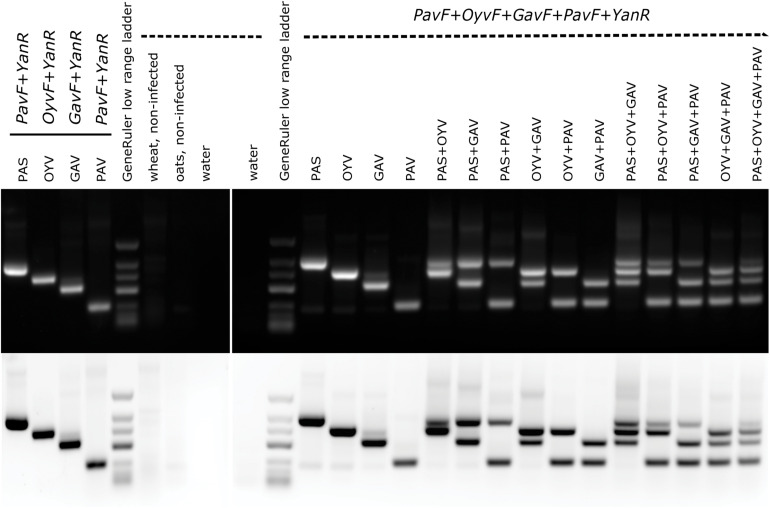
Single and multiplex RT-PCR detection of BYDV-PAS, -OYV, -GAV, and -PAV. The expected RT-PCR product sizes are as follows: BYDV-PAS 476 bp, BYDV-OYV 401 bp, BYDV-GAV 317 bp, and BYDV-PAV 193 bp. GeneRuler low range DNA ladder (ThermoScientific) was used as a size marker (700/500/400/300/200/150/100 bp). 1 μg of total RNA extractions from field-derived plant samples was used as a template in cDNA synthesis reaction. For PCR step, artificial mixes of the first-strand synthesis products were mixed in equal quantities (0.5 μl of each template). The following isolates were used: BYDV-PAS Imavere, BYDV-OYV Avinurme2, BYDV-GAV Kumna, and BYDV-PAV Olustvere1-B.

A multiplex-RT-PCR specifically targeting BYDV-PAS, BYDV-OYV, BYDV-GAV, and BYDV-PAV found in this study was established for simultaneous monitoring. The single sets and the multiplex primer set produced RT-PCR fragments with the expected sizes: 476 bp for BYDV-PAS, 401 bp for BYDV-OYV, 317 bp for BYDV-GAV, and 193 bp for BYDV-PAV (Figure 4). Simultaneous detection of one, two, three, or four different targets in a single amplification was achieved, although the intensity of the bands corresponding to different viruses varied a bit if multiple templates were present in a single reaction (Figure 4). The reason might be a result of unbalanced template amounts as the real amount of viral template in each sample was unknown (the first-strand synthesis products of the previously used samples were artificially mixed to have different templates in one reaction). Weak non-specific signals were detected in the background when the picture was studied in inverted-color mode.

### *In silico* Primer Analysis

The sensitivity and the specificity of the primers were estimated for 32 primer pairs, including the primer pairs designed in this study, are included in the Supplementary Tables 1A–D. The sensitivity reflects the ability of the primer pair to bind correctly to its target sequences, and the specificity measures the ability of the primer pair not binding to the non-target sequences.

Regarding the new candidate species BYDV-OYV, the novel “OyvF & Yan-R” primer pair shows high sensitivity and specificity. Regarding the BYDV-PAS isolates, both the primer pairs “PasF & Yan-R” and “PASF & PASR” show high specificity. The primer pair “GavF & Yan-R” shows the best complementary to BYDV-GAV isolates whereas the primer pair “MAVF & MAVR” shows the highest sensitivity and specificity to BYDV-MAV subspecies solely. The primer pairs “PavF & Yan-R” and “PAVL1 & PAVR1” show the highest sensitivity and specificity for BYDV-PAV isolates, although these primer pairs may miss and fail to detect several isolates. Theoretically, if three mismatches are allowed, “PavF & Yan-R” shows possible annealing to the BYDV-PAS isolates. However, this was not noticed in our multiplex-RT-PCR (Figure 4). The primer pair “F3-PAV & B3-PAV” is the only primer pair targeting BYDV-PAV-CN isolates (although not all of them) while retaining a high specificity.

Several analyzed primer pairs show potential unspecific amplification for isolates belonging to different species. For example, the primer pair “PAV (forward) & PAV (reverse)” does not discriminate BYDV-PAV, BYDV-PAS, and BYDV-MAV/GAV; the primer pair “MAVL1 & MAVR1” does not discriminate BYDV-MAV and BYDV-PAV, and “MAVF2 & Yan-R” recognizes BYDV-GAV instead of BYDV-MAV even if no mismatches to the target are allowed. If mismatches are allowed, the probability of unspecific recognition also shows up for several other primer pairs.

Among the universal primers designed to recognize multiple BYDV species, the primer pair “Luteo1F & YanR-New” is the most universal — theoretically, it only fails to detect BYDV-KerII and -KerIII isolates, two isolates of BYDV-PAV and one BYDV-PAV-CN isolate.

## Discussion

By high throughput sequencing of field samples from a country-wide survey in Estonia, we identified five species of cereal/barley YDVs – CYDV-RPS, BYDV-GAV, BYDV-PAS, BYDV-PAV, and BYDV-OYV which are described in the current study. The putative novel luteovirus, BYDV-OYV, was also identified in the samples of meadow fescue collected from Sweden and included in our analysis. The same survey identified brome mosaic virus, European wheat striate mosaic virus, oat sterile dwarf virus, and putative cereal closterovirus (Sõmera et al., 2016, 2020; Sõmera et al., unpublished). In addition, wheat dwarf virus was found in 2017 (Sõmera et al., 2019).

In the past, the novel luteovirus BYDV-OYV was detected on one occasion from a neighboring country, Latvia. During this previous study, a 502-nt fragment of the CP gene was amplified and sequenced using the luteovirus universal primer pair Lu1/Lu4. A phylogenetic relationship to a group of Chinese isolates of BYDV-PAV (BYDV-PAV-CN) was proposed but the taxonomic status of BYDV-OYV and BYDV-PAV-CN remained unclear (Bisnieks et al., 2004).

After the start of the sequencing era, it was suggested that the isolates of BYDV-PAV-like sequences be allocated to three quasi-descriptive groupings: PAV-I, PAV-II, and PAV-III corresponding to BYDV-PAV, BYDV-PAS, and BYDV-PAV-CN (Liu et al., 2007). BYDV-PAS, which was initially recognized as the severe strain of BYDV-PAV breaking the standard tolerance in oats (Chay et al., 1996), has now been recognized as a distinct species due to genome sequence divergences. Additional phylogenetic analyses have indicated that BYDV-PAV and BYDV-PAV-CN isolates also split into two distinct major groups (Miller et al., 2002; Boulila, 2011; Wu et al., 2011). Sequencing the complete genome of BYDV-PAV-CN confirmed that BYDV-PAV-CN fullfills the species demarcation criteria of sequence divergence established for the genus *Luteovirus*. However, the authors suggested waiting until the genome of BYDV-OYV is sequenced to determine whether these two viruses could be classified as a single species or two distinct species (Liu et al., 2007).

Extensive recombination detection analyses indicate that recombination imprints are common in BYDV genomes (Pagan and Holmes, 2010; Wu et al., 2011). The existence of recombination events further complicates the taxonomy of BYDV species. When the viral RdRp genes have been analyzed, it has been observed that the isolates of BYDV-PAV and BYDV-MAV were present in a monophyletic clade whereas BYDV-PAV-CN formed another monophyletic lineage together with the isolates of BYDV-PAS. At the same time, the phylogenies of the CP and MP genes placed BYDV-PAV and BYDV-PAS isolates in a monophyletic clade separate from BYDV-MAV and BYDV-CN-PAV (Miller et al., 2002; Hall, 2006). The characterization of the BYDV-OYV CP gene suggested that there is at least one more putative BYDV species close to BYDV-PAV-CN (Bisnieks et al., 2004). In the current study, we present for the first time the complete genome characterization of seven BYDV-OYV isolates from Estonia, and a near-complete genome sequence for one isolate from Sweden.

Our phylogenetic analyses and sequence alignment studies indicate that BYDV-PAV-CN and BYDV-OYV share the closest relationships between their 3′ gene blocks (CP-RTD/MP), but slightly looser relationships between their 5′ gene blocks (P1/RdRp), which instead show the closest relationships between BYDV-PAS and BYDV-OYV. Remarkably, a very highly conserved region (also known as ORF3a) resides between these two gene blocks. Most probably, such a highly conserved region is a suitable template for copy-choice recombination during mixed infection. The template-switching model includes dissociation of the replicase and nascent strand, followed by nascent RNA strand hybridization to the region of complementarity in the acceptor strand. It has been proposed that the subgenomic promoters at the end of the 5′gene block (within the region encoding RdRp) of luteoviruses serve as the “hot spot” sites for recombination during replication (Miller et al., 1995; Koev et al., 1999). Alternatively, a bulged stem–loop of the sgRNA promoter region in the acceptor strand may act as an interactor, which helps the replicase (or a host factor involved in the replication complex) to relocate during a template-switching process near the complementary region (Miller and Koev, 1998). In recombination detection analyses, BYDV-OYV was identified as a putative minor parent for recombinant regions in the genomes of BYDV-PAV-CN and BYDV-PAS. Agreeing with the sequence comparisons, the recombination analyses suggest that the common PAS genotype (represented by Imavere isolate) recombined with BYDV-OYV leading to a more diverse BYDV-PAS-129 now having a part of ORF5 from BYDV-OYV. Furthermore, BYDV-PAS-129 recombined with BYDV-OYV resulting in BYDV-PAV-CN which then has ORF3/4 from BYDV-PAS-129 and the end part of ORF5 from BYDV-OYV.

According to the identity calculations, the multiple gene products of BYDV-OYV show differences of around 10% or higher when compared with the respective gene products of other BYDVs (Table 3). Species demarcation criteria in the genus *Luteovirus* is a >10% difference in amino acid sequence identity of any gene product from its closest relative. Therefore, considering the sequence analyses described and taking into account that both viruses have been found from several different locations and years, we propose creating two new species in the genus *Luteovirus*: BYDV-OYV and BYDV-PAV-CN.

Based on these taxonomic distinctions, the incidence results can be analyzed. In our survey, BYDV-PAS and BYDV-OYV were two YDVs whose incidence in the sampled plants was higher compared to other YDV species (BYDV-PAV, BYDV-GAV, and CYDV-RPS). Interestingly, BYDV-PAV was found only in one sample. The absence of BYDV-PAV, but the abundance of BYDV-PAS, has been reported in Alaska, which is exposed to climatic conditions similar to Northern Europe with long photoperiods and cooler temperatures during the growing season (Robertson and French, 2007). It has been speculated that this situation is related to the ability of BYDV-PAS to overwinter in perennial grasses. In the Czech Republic, where BYDV-PAS is more prevalent than BYDV-PAV, this difference has been suggested to be related to the number of aphid species capable of transmitting these viruses – BYDV-PAV is recorded as being transmitted by *Rhopalosiphum padi* and *Sitobion avenae* whereas BYDV-PAS is also transmitted by *R. maidis* and *Metopolophium dirhodium* (Jarošová et al., 2013).

Whereas BYDV-PAS was most commonly identified from winter wheat (although it was also present in some samples of spring wheat and other cereals) in our survey, BYDV-OYV was detected from oats and spring wheat. The first discovery of BYDV-OYV was also in oats (Bisnieks et al., 2004). Our finding of BYDV-OYV infection in meadow fescue in Sweden indicates one possible overwintering host. The occurrence of BYDV-OYV in spring cereals suggests that it might be transmitted from perennial grasses to cereals during the growing season whereas BYDV-PAS might be transmitted to winter cereals during the autumn flight of the aphids, and later on, to the spring cereals. Intriguingly, differences in identified host species may account for differences in dominant vector species. The aphid vector of BYDV-OYV is currently unknown and needs to be revealed.

In the case of cereal-infecting YDVs, BYDV-PAV, BYDV-MAV, and MYDV-RMV are generally accepted as being the most prevalent YDV species worldwide (Signoret and Maroquin, 1990; Zhou and Zhang, 1990; Henry and Adams, 2003; Parry et al., 2012). The raised CP-specific antisera can detect the serotypes of CYDV-RPV, MYDV-RMV, BYDV-PAV, BYDV-MAV, and BYDV-SGV, which are the five most characteristic YDVs in the United States (Gildow, 1990). Similarly, the most widely used multiplex-RT-PCR protocol enables one to discriminate against these viruses (Malmstrom and Shu, 2004). The sequencing of virus amplicons has revealed that BYDV-PAS isolates could have been incorrectly identified as BYDV-PAV (Robertson and French, 2007). Therefore, the original protocol was improved to discriminate BYDV-PAS by adding a restriction digestion step (Kundu et al., 2009) or an extra pair of primers for BYDV-PAS (Laney et al., 2018). Even if sequenced, false identification of new BYDV-PAS isolates as BYDV-PAV can easily occur if only BYDV-PAV sequences are retrieved from GenBank and included in phylogenetic analyses as a subset of the genome and CP gene sequences of BYDV-PAS isolates still exist under the name of BYDV-PAV there (see Najar et al., 2017).

The existing B/CYDV antisera fail to detect or do not allow proper discrimination between some species and may lead to biased epidemiological analysis. Earlier studies have noticed that BYDV-PAV antiserum does not discriminate BYDV-PAV and BYDV-PAS (Chay et al., 1996). Likewise, BYDV-OYV was detected by BYDV-PAV antiserum (Bisnieks et al., 2004), and BYDV-KerII and BYDV-KerIII were detected by BYDV-PAV and BYDV-MAV antisera (Svanella-Dumas et al., 2013). BYDV-GPV, a tentative member of the genus *Polerovirus* that has been known to not react with antisera raised against MAV, PAV, SGV, RPV, or RMV and has exclusively been detected in China until recently (Zhang et al., 2009), was suddenly reported to occur in the Czech Republic according to HTS data analysis (Singh et al., 2020).

The occurrence of other species might also be underestimated. For example, another YDV species found in our study, CYDV-RPS, can also be masked by cross-reaction of CYDV-RPV antiserum (no CYDV-RPS specific antiserum has been raised) or amplified by CYDV-RPV primers. According to currently existing data, CYDV-RPS seems to be very rare in comparison to CYDV-RPV although both are transmitted by *R. padi*. There are only a few earlier findings of CYDV-RPS, all based on sequencing – the first ones from Mexico (Miller et al., 2002) and Iran (Rastgou et al., 2005) were detected by RT-PCR using CYDV-RPV primers, and all the recent ones come from HTS analyses performed in the United Kingdom (Pallett et al., 2010), the United States (Malmstrom et al., 2017), the Czech Republic (Singh et al., 2020), and Estonia (this study). Similar to the previous example, there is a lack of specific antisera for BYDV-GAV and it can be misidentified as BYDV-MAV due to cross-reaction of MAV-antiserum (Zhou and Zhang, 1990). For a long time, BYDV-GAV was supposed to be exclusively spread in China (Liu et al., 2007), but recent sequencing-related findings confirm its presence in Poland (Trzmeil, 2017) and in Estonia (this study). Therefore, further findings of BYDV-GAV may be expected in other regions in Eurasia as well when the detection will be verified by sequencing. It has been recorded that BYDV-GAV found from China is transmitted by *S. avenae* and *Schizaphis graminum* whereas the master species BYDV-MAV collected from North America is known not to be transmitted by *S. graminum* (Rochow, 1982; Liu et al., 2007). The biological characterization of European BYDV-GAV isolates remains to be carried out. Finally, recent HTS-data based identifications of new cereal-infecting poleroviruses, MaYMV, and BVG related to MYDV-RMV (Chen et al., 2016; Zhao et al., 2016), lead to the question whether these viruses could react with MYDV-RMV antibodies and might have been interpreted as MYDV-RMV in previous serotype-specific MYDV-RMV identifications.

The use of antibody detection or multiplex RT-PCR for simultaneous amplification of different virus templates is helpful to reduce diagnostic costs (several multiplex-RT-PCR protocols are available for YDVs, see: Malmstrom and Shu, 2004; Deb and Anderson, 2008; Tao et al., 2012). However, these multiplex tests have been designed to discriminate the species characteristic for the geographic region of sampling and are based on a limited set of YDV genetic diversity in the databases. Therefore, they should be used with care as sequences of other YDV species can be amplified and misidentified by the primers used in these protocols, which remains unknown if the PCR product is not sequenced. In addition, other YDV species different enough in primer annealing positions may remain unidentified as suggested by our *in silico* primer binding test and the similar analysis carried out by Laney et al. (2018). The potential to detect a subset of BYDV species as well as the specificity and sensitivity of commonly used primer pairs varied greatly (Supplementary Table 1). Therefore, it can be concluded that the ability of a diagnostician to detect the virus(es) infecting a sample relies on the techniques used and their specificity.

HTS technologies can provide full virological indexing of samples while being as sensitive as RT-PCR (Santala and Valkonen, 2018), although they still represent a much higher cost per sample. Nevertheless, their use in virus surveys, alone or in combination with targeted RT-PCR, will most probably grow in the future. Detection of new virus species and genotypes by HTS indicates a growing need to adapt the detection primers continuously and make the process efficient and quick (Katsiani et al., 2018). With a significant impact on primer development, HTS technologies are helping to target the existing diversity of the viral population within ecosystems and are taking steps toward an improved understanding of virus epidemiology and evolution, including that of cereal and barley YDVs.

## Data Availability Statement

The datasets presented in this study can be found in online repositories. The names of the repository/repositories and accession number(s) can be found below: https://www.ncbi.nlm.nih.gov/genbank/, MK012642–MK012664.

## Author Contributions

MS designed the study, prepared the HTS libraries and analyzed the data, performed the multiplex-RT-PCR experiment, wrote the manuscript, and prepared the figures and tables. SM advised on HTS data analysis and took part in planning and finalizing the manuscript. LT performed *in silico* primer analysis and discussed the data. PS performed the field sampling and collected metadata about the sampled sites. KS sequenced the Swedish isolate of BYDV-OYV. AK supervised KS, performed recombination detection analysis, discussed the interpretation of the data, and took part in finalizing the manuscript. All authors agreed to be accountable for the content of the work.

## Conflict of Interest

The authors declare that the research was conducted in the absence of any commercial or financial relationships that could be construed as a potential conflict of interest.
