# Corrigendum: A Survey Using High-Throughput Sequencing Suggests That the Diversity of Cereal and Barley Yellow Dwarf Viruses Is Underestimated

**DOI:** 10.3389/fmicb.2021.772637

**Published:** 2021-11-19

**Authors:** Merike Sõmera, Sébastien Massart, Lucie Tamisier, Pille Sooväli, Kanitha Sathees, Anders Kvarnheden

**Affiliations:** ^1^Department of Chemistry and Biotechnology, Tallinn University of Technology, Tallinn, Estonia; ^2^Laboratory of Integrated and Urban Phytopathology, Gembloux Agro-Bio Tech – University of Liège, Gembloux, Belgium; ^3^Department of Plant Protection, Estonian Crop Research Institute, Jõgeva, Estonia; ^4^Department of Plant Biology, Swedish University of Agricultural Sciences, Uppsala, Sweden; ^5^Institute of Agricultural and Environmental Sciences, Estonian University of Life Sciences, Tartu, Estonia

**Keywords:** HTS, *Luteovirus*, BYDV, CYDV, diagnostics, wheat, epidemiology, OYV

In the original article, there was a typographical mistake in Table 1 as published. The sequence of Yan-R primer should read “TGTTGAGGAGTCTACCTATTTG”. The Yan-R primer sequence was given correct in the Supplementary Table 2. The correct Yan-R primer was used in experiments undertaken in this study and thus it does not impact on validity of the results. The corrected Table 1 appears below.

**Table 1:** Primers used in the current study.

**Primers**	**Nucleotide sequence 5' to 3'**	**Expected position**	**References**
P326(+)	GACTTCGAGGCNGANCTCGCT	330–350	Liu et al., 2007
P12(-)	GCTCCGTCTGTGACCGCAAT	1226–1245	Liu et al., 2007
P115(+)	GGGTTTTTAGAGGGGCTCTGT	1157–1177	Liu et al., 2007
OYV5(-)	TCACCATGTTGAAGCCGTATT	2199–2219	This study
OYV4(+)	ATGTTCGTTGAGGATAAGATGC	1973–1994	This study
OYV1(-)	AGTACGTGAGAGCTAATGTAC	2800–2820	This study
Shu-F	TACGGTAAGTGCCCAACTCC	2650–2669	Malmstrom and Shu, 2004
Yan-R	TGTTGAGGAGTCTACCTATTTG	3459–3480	Malmstrom and Shu, 2004
OYV2(+)	TGAACTCGACACTGCGTGCA	3237–3256	This study
OYV3(-)	CTACCCGAGCTTATGAACCT	4857–4876	This study
P47(+)	GCAAAGGAGTACAAGGCACAAT	4777–4798	Liu et al., 2007
P55(-)	GGATTGCTATGGTTTATGTCC	5491–5511	Liu et al., 2007
OYV5pR1	AAGTCCGTCCAAGCCTCGG	533–541	This study
OYV5pR2	CTTTGACGCTGGCTCCAATGAGC	161–183	This study
PasF	GAAGAGGGCCAAATTCTATACC	3003–3024^*^	This study
OyvF	CCAATTCCTCAGGGATCC	3080–3097	This study
GavF	GTTACAAGATCACAAACGTCAAG	3156–3178^**^	This study
PavF	CTTCACAATCAGCAGGAC	3261–3278^***^	This study

*Primer binding positions are shown respective to full length sequence of BYDV-OYV Avinurme2 isolate (MK012645; unless mentioned otherwise)*.

* *BYDV-PAS (MK012660)*,

** *BYDV-GAV (MK012663), and*

*** *BYDV-PAV (MK012661)*.

The authors apologize for this error and state that this does not change the scientific conclusions of the article in any way. The original article has been updated.

## Publisher's Note

All claims expressed in this article are solely those of the authors and do not necessarily represent those of their affiliated organizations, or those of the publisher, the editors and the reviewers. Any product that may be evaluated in this article, or claim that may be made by its manufacturer, is not guaranteed or endorsed by the publisher.
