# Supplementary material for: A Survey Using High-Throughput Sequencing Suggests That the Diversity of Cereal and Barley Yellow Dwarf Viruses Is Underestimated
Source: Front Microbiol. 2021 May 11;12:673218. doi: 10.3389/fmicb.2021.673218 (PMC8144474; doi:10.3389/fmicb.2021.673218)
Supplement: Supplementary file 2 [file Table_2.docx]

**Supplementary Table S2.** Virus-specific pairs of primer used for *in silico* binding tests. Primer pairs designed in this study are indicated in gray background color.

| **Target virus** | **Primer** | **Sequence (5’ to 3’)** | **Reference** |
| --- | --- | --- | --- |
| **BYDVs** | Lu 1 | CCAGTGGTTRTGGTC | Robertson *et al .*, 1991 |
|  | Lu 4 | GTCTACCTATTTGG | Robertson *et al.*, 1991 |
|  |  |  |  |
|  | Shu-F | TACGGTAAGTGCCCAACTCC | Malmstrom and Shu, 2004 |
|  | Yan-R | TGTTGAGGAGTCTACCTATTTG | Malmstrom and Shu, 2004 |
|  |  |  |  |
|  | Luteo1F | TTCGGMSARTGGTTGTGGTCCA | Svanella-Dumas et al., 2013 |
|  | YanR-New | TGTTGAGGAGTCTACCTATTTG | Svanella-Dumas et al., 2013 |
| **BYDV-GAV** | F3-GAV | AGTGAACAGAGCACCATC | Zhao *et al.*, 2010 |
|  | B3-GAV | GGCTTTGTACTCCTTAGCT | Zhao *et al.*, 2010 |
|  |  |  |  |
|  | GAV1 | GTAGAAATAACCGCAGGAG | Tao *et al.*, 2012 |
|  | GAV2 | GACTTGAGTATTCCACCTGA | Tao *et al.*, 2012 |
|  |  |  |  |
|  | GavF | GTTACAAGATCACAAACGTCAAG | This study |
|  | Yan-R | TGTTGAGGAGTCTACCTATTTG | Malmstrom and Shu, 2004 |
| **BYDV-MAV** | MAV2-F | AATAACCGCAGGAGAAATGG | Malmstrom and Shu, 2004 |
|  | Yan-R | TGTTGAGGAGTCTACCTATTTG | Malmstrom and Shu, 2004 |
|  |  |  |  |
|  |  |  |  |
|  | MAVL1 | CAACGCTTAACGCAGATGAA | Deb and Anderson, 2008 |
|  | MAVR1 | AGGACTCTGCAGCACCATCT | Deb and Anderson, 2008 |
|  |  |  |  |
|  | MAVF | GCATGGTGGAACTCCAACAACAGT | Laney *et al.*, 2018 |
|  | MAVR | AGGTAAAACTTGGGATCAGCACCCCTA | Laney *et al.*, 2018 |
| **BYDV-OVY** | OyvF | CCAATTCCTCAGGGATCC | This study |
|  | Yan-R | TGTTGAGGAGTCTACCTATTTG | Malmstrom and Shu, 2004 |
| **BYDV-PAS** | PasF | GAAGAGGGCCAAATTCTATACC | This study |
|  | Yan-R | TGTTGAGGAGTCTACCTATTTG | Malmstrom and Shu, 2004 |
|  |  |  |  |
|  | PASF | GGAGACGACTGTGTCATCATCACTGAG | Laney *et al.*, 2018 |
|  | PASR | TGTCGTTTGTGATAGGTGTCTCC | Laney *et al.*, 2018 |
| **BYDV-PAV** | PAV (forward) | AATGCCCAGCGCTTTCAG | Balaji *et al.*, 2003 |
|  | PAV (reverse) | GCGGACGCGTGTGACTTAA | Balaji *et al.*, 2003 |
|  |  |  |  |
|  | PAV-R | ACCTAGACGCGCAAATCAAA | Malmstrom and Shu, 2004 |
|  | Yan-R | TGTTGAGGAGTCTACCTATTTG | Malmstrom and Shu, 2004 |
|  | PAV- F | AGGACCTAGACGCGCAAA | Nagy *et al.*, 2006 |
|  | PAV-R | GGTTCCATTGGCCTTGTAGA | Nagy *et al.*, 2006 |
|  |  |  |  |
|  | PAVL1 | AGAGGAGGGGCAAATCCTGT | Deb and Anderson, 2008 |
|  | PAVR1 | ATTGTGAAGGAATTAATGTA | Deb and Anderson, 2008 |
|  |  |  |  |
|  | F3-PAV | CAACTATGTTGTCTCGTATGGA | Zhao *et al.*, 2010 |
|  | B3-PAV | TGGTCAGTTATGGTGGAAAG | Zhao *et al.*, 2010 |
|  |  |  |  |
|  | PAV1 | ATGAATTCAGTAGGCCGTAGG | Tao *et al.*, 2012 |
|  | PAV2 | CTATTTGGCCGTCATCAAGTG | Tao *et al.*, 2012 |
|  |  |  |  |
|  | BYDV-P5-fw | ACTTGGAACATACCAGGGACAG | Svanella-Dumas *et al.*, 2013 |
|  | BYDV-3'NC-rev2 | GTCTTCAATCCTGACGATCGG | Svanella-Dumas *et al.*, 2013 |
|  |  |  |  |
|  | PavF | CTTCACAATCAGCAGGAC | This study |
|  | Yan-R | TGTTGAGGAGTCTACCTATTTG | Malmstrom and Shu, 2004 |
| **MYDV-RMV** | S2b-F | TCACCTTCGGGGCGTCTCTTTCTG | Malmstrom and Shu, 2004 |
|  | Yan-R | TGTTGAGGAGTCTACCTATTTG | Malmstrom and Shu, 2004 |
|  |  |  |  |
|  | RMV-F | CGTGAATGAATACGGGAGGT | Nag*y et al.*, 2006 |
|  | RMV-R | CCTATTTGGGGTTTTGAACA | Na*gy et al.,* 2006 |
|  |  |  |  |
|  | RMVL1 | GACGAGGACGACGACCAAGTGGA | Deb and Anderson, 2008 |
|  | RMV R | GCCATACTCCACCTCCGATT | Deb and Anderson, 2008 |
|  |  |  |  |
|  | RMV-MTF | TCGAAGGACTCTCTCACGGGCAAT | Lan*ey et al.*, 2018 |
|  | RMV-MTR | AGAGGCCTCGGAGATGAACTCCAAAG | Laney *et al.*, 2018 |
| **CYDV-RPV** | RPV (forward) | ACGAGTTGGACCCCCATTG | B*alaji et al.*, 2003 |
|  | RPV (reverse) | GATCATCTTCGCTGGGAAGCT | Balaji *et al.*, 2003 |
|  |  |  |  |
|  | S2a-F | TCACCTTCGGGCCGTCTCTATCAG | Malmstrom and Shu, 2004 |
|  | Yan-R | TGTTGAGGAGTCTACCTATTTG | Malmstrom and Shu, 2004 |
|  |  |  |  |
|  | RPV L | ATGTTGTACCGCTTGATCCAC | Deb and Anderson, 2007 |
|  | RPV R | GCGAACCATTGCCATTG | Deb and Anderson, 2007 |
|  |  |  |  |
|  | RPV L | ATGTTGTACCGCTTGATCCAC | Deb and Anderson, 2007 |
|  | RPVR | CGGCTAGTTTTGTGCTCAGTTTAG | Laney *et al.*, 2018 |
| **BYDV-SGV** | Shu-F | TACGGTAAGTGCCCAACTCC | Malmstrom and Shu, 2004 |
|  | SGV-R1 | ACATTTCTTCGTGTGTTGCG | Malmstrom and Shu, 2004 |
|  |  |  |  |
|  | Shu-F | TACGGTAAGTGCCCAACTCC | Malmstrom and Shu, 2004 |
|  | SGV-R2 | ACATTTTTGCGTGCGTTGCG | Malmstrom and Shu 2004 |
|  |  |  |  |
|  | SGV L2 | ACCAGATCTTAGCCGGGTTT | Deb and Anderson, 2008 |
|  | SGV R2 | CTGGACGTCGACCATTTCTT | Deb and Anderson, 2008 |
|  |  |  |  |
|  | SGVF | CACATCTGCAATCAATCCTCCTCTCA | Laney *et al.*, 2018 |
|  | SGV-R1 | ACATTTCTTCGTGTGTTGCG | Malmstrom and Shu, 2004 |
